# Amniotic Epithelial Cells from the Human Placenta Potently Suppress a Mouse Model of Multiple Sclerosis

**DOI:** 10.1371/journal.pone.0035758

**Published:** 2012-04-26

**Authors:** Yu Han Liu, Vijesh Vaghjiani, Jing Yang Tee, Kelly To, Peng Cui, Ding Yuan Oh, Ursula Manuelpillai, Ban-Hock Toh, James Chan

**Affiliations:** 1 Centre for Inflammatory Diseases, Department of Medicine, Monash University, Clayton, Victoria, Australia; 2 Center for Reproduction and Development, Monash Institute of Medical Research, Monash University, Clayton, Victoria, Australia,; University Hospital Jena, Germany

## Abstract

Human amniotic epithelial cells (hAEC) have stem cell-like features and immunomodulatory properties. Here we show that hAEC significantly suppressed splenocyte proliferation *in vitro* and potently attenuated a mouse model of multiple sclerosis (MS). Central nervous system (CNS) CD3^+^ T cell and F4/80^+^ monocyte/macrophage infiltration and demyelination were significantly reduced with hAEC treatment. Besides the known secretion of prostaglandin E2 (PGE2), we report the novel finding that hAEC utilize transforming growth factor-β (TGF-β) for immunosuppression. Neutralization of TGF-β or PGE2 in splenocyte proliferation assays significantly reduced hAEC-induced suppression. Splenocytes from hAEC-treated mice showed a Th2 cytokine shift with significantly elevated IL-5 production. While transferred CFSE-labeled hAEC could be detected in the lung, none were identified in the CNS or in lymphoid organs. This is the first report documenting the therapeutic effect of hAEC in a MS-like model and suggest that hAEC may have potential for use as therapy for MS.

## Introduction

Multiple sclerosis (MS) is a T cell-mediated demyelinating disease affecting over two million people worldwide with no cure available [1], [2]. Myelin oligodendrocyte glycoprotein (MOG) [3], [4] induced experimental autoimmune encephalomyelitis (EAE) is an animal model extensively used to study the pathogenesis of MS by inducing paralytic symptoms, and demyelination in the CNS accompanied by perivascular mononuclear cell infiltration [5], [6], [7].

Mesenchymal stem (stromal) cells, which can inhibit T cell expansion, are being trialed as a therapy for MS [8]. We explored the potential of human amniotic epithelial cells (hAEC) to suppress a mouse model of MOG-induced EAE. hAEC originate from pluripotent embryonic epiblasts, express some embryonic and mesenchymal stem cell markers [9], [10], [11], [12], and are isolated from the amniotic membrane of the human placenta. hAEC can be obtained in large amounts without extended *ex vivo* expansion or ethical concerns compared to bone marrow and embryo derived stem cells. They have stem cell-like features and can differentiate into lineages representing cells originating from the three germ layers [10], [11], and express low levels of Class IA human leukocyte antigens (HLA) and lack Class II antigens which may potentially reduce the risk of immune-rejection after transplantation [10], [12]. Previous studies have shown that hAEC also have immunomodulatory properties and inhibit mixed lymphocyte reactions and mitogen stimulated T cell proliferation [13], [14] where some of these effects may be attributed by secreted factor(s) [15]. Besides having effect on T cells, hAEC have been shown to secrete neurotrophic substances [16], [17] suggesting that hAEC transplantation may be useful for the treatment and repair of inflammatory neurological diseases. Overall, the ease of accessibility, low antigenicity, repair capacity and immunomodulatory properties make hAEC an important cell type for regenerative medicine.

Here, we show that intravenous hAEC transplantation potently ameliorated MOG-induced EAE, significantly reduced CD3^+^ T cells and F4/80^+^ monocyte/macrophage infiltration and demyelination within the central nervous system (CNS). We also showed that hAEC secreted transforming growth factor-β (TGF-β) and prostaglandin E2 (PGE2) in primary culture. Blocking TGF-β using a neutralizing antibody or PGE2 by indomethacin significantly reduced the suppression of splenocyte proliferation by hAEC. In addition, splenocytes from hAEC-treated mice produced significantly more Th2 cytokine IL-5 compared to control. Injected CFSE-labeled hAEC were detected in the lung but none were detectable in the CNS or peripheral lymphoid organs. We suggest that hAEC may have potential for treating MS due to their immunosuppressive effects and improvement seen within the CNS of the mouse model of MS.

## Materials and Methods

### Ethics Statement

The study was approved by Southern Health Human Research Ethics Committee and the Institutional Review Board of Monash University. Informed, written consent was obtained from each patient prior to amnion membrane collection. Tissues were retrieved from placentae delivered by healthy women with a normal singleton pregnancy undergoing elective cesarean section at term (37–40 weeks gestation; n = 30). Animal experimentation was approved by the Animal Ethics Committee, Monash University (approval number MMCB 2009/16).

### hAEC isolation and culture

Cell isolation, culture and characterization were as described previously [10], [18]. Briefly, amnion membranes were cut into small pieces and digested twice in 0.05% trypsin:EDTA (Gibco) for 40 min at 37°C. Following inactivation of trypsin with newborn calf serum, dispersed cells were washed in DMEM/F12 medium (Gibco) and erythrocytes lysed in hypotonic solution. Batches (n = 15) >99% positive for the epithelial markers cytokeratin-7 and 8/18 (Dako, Denmark) by flow cytometry and displaying a cobblestone epithelial morphology in culture were used for *in vivo* and *in vitro* studies [19].

### EAE induction and treatment

EAE was induced in female C57BL/6 mice 8–12 weeks old by immunization of 200 µg MOG_35–55_ peptide (GL Biochem, China) and 350 ng pertussis toxin (Sigma-Aldrich) given on the day of immunization and repeated 2 days later [7]. MOG_35–55_ peptide was emulsified with 100 µl complete Freud's antigen (CFA; Sigma-Alderich) and 4 mg/ml *Mycobacerium tuberculosis* (Difco Laboratories) in PBS. Two million hAEC (primary cells, passage 0, pooled from 4 donors) in basal media were administered intravenously on day 9 after immunization in 3 independent experiments, while control littermates remained untreated (hAEC-treated mice, n = 25; EAE control mice, n = 26, in total 3 experiments). Animals were monitored daily and neurological impairment scored on an arbitrary clinical score: 0, no clinical sign; 1, limp tail; 2, limp tail and hind limb weakness; 3, severe hind limb paresis; 4, complete hind limb paresis; 5, moribund or death [6], [7]. All studies were performed with approval of the local animal ethics committee. As required by animal ethics, mice were euthanized upon reaching a score of 3.

### Detection of immunoregulatory molecules produced by hAEC

hAEC (n = 7 donors) were cultured for 7 days in complete DMEM-F12 and then 2 days more in serum-deprived DMEM-F12 medium. TGF-β1, interleukin-10 (IL-10) and hepatocyte growth factor (HGF) were measured using ELISA kits (R&D Systems). Nitric oxide (NO) production was detected using Griess reagent system (Promega). PGE2 was detected by EIA kit (Cayman Chemical). Cytokine and PGE2 measurements are expressed as pg/ml/million hAEC.

### Splenocyte suppression assays and cytokine detection

Mouse splenocytes were used as responders for mitogen/antigen stimulation. Gamma-irradiated hAEC (20 Gy) as suppressor cells were co-cultured at different ratios with splenocytes (5×10^5^) in 96-well plates. For antigen non-specific suppression, splenocytes from naïve mice were stimulated with the mitogen ConA (5 or 10 µg/ml, Sigma-Aldrich). For antigen-specific suppression, splenocytes from EAE-induced mice were stimulated with MOG_35–55_ peptide (1, 10, or 100 µg/ml). Proliferation was assessed by ^3^H-thymidine incorporation as described earlier [6]. Blocking assays were performed by adding anti-human TGF-β monoclonal antibody (0.4 µg/ml, R&D Systems) or the PGE2 inhibitor indomethacin (1 nM, Sigma-Aldrich) to hAEC/splenocyte cultures. In some experiments, 72 hr culture supernatants were collected for mouse cytokine detection (IL-4, IL-5, IL-10, IL-17, IFN-γ, GM-CSF, TNF-α) by flow cytometry according to the manufacturer's instructions (Bender MedSystems/FlowCytomix, eBioscience).

### Detection of anti-MOG antibodies

Anti-MOG antibodies were detected in mouse sera as described previously [6]. Sera were collected from mice at the end-point of *in vivo* experiments, and tested at 1∶300 dilutions in 96-well plates coated with 5 µg/ml of MOG_35–55_ peptide. Anti-MOG antibodies bound to MOG were detected by horseradish-peroxidase conjugated goat anti-mouse IgG (Dako, Denmark) and then developed by TMB ELISA substrate (Thermo Scientific). Mean absorbance of samples analyzed in triplicate was calculated minus values from uncoated controls using VICTORE X Multilabel Counter (Perkin-Elmer).

### Flow cytometry

Cells from spleen and inguinal lymph nodes which were harvested from hAEC-treated and EAE control mice were stained for 30 min at 4°C with PB-, PE-, or APC-conjugated monoclonal antibodies: CD4 (clone RM4–5, 1 µg/ml, BD), CD8 (clone 53-6.7, 1 µg/ml, BD), CD19 (clone 1D3, 0.25 µg/ml, eBioscience), CD25 (clone not specified, 1 µg/ml, BD), Foxp3 (clone FJK-16s, 1 µg/ml, APC anti-mouse/rat staining kit, eBioscience), or matched isotype control IgG (BD or eBioscience). The percentage of stained cells was analyzed by CANTO flow cytometer (BD). Fc receptors of splenocytes were blocked prior to antibody staining by anti-mouse CD16/CD32 (clone 2.4G2, 2.5 µg/ml, BD) for at least 15 min at 4°C.

### Histology

Histological assessment of spinal cord was performed as previously described [6]. On average, 20 sections (5 µm) taken 20 µm apart from each mouse were examined. The extent of inflammation and demyelination was evaluated blinded with hematoxylin and eosin (H&E) and Luxol fast blue (LFB) stains, respectively [6], [7]. For inflammation, evaluation was performed using H&E-stained sections and scored as follows: 0, no inflammation; 1, cellular infiltrate only in the perivascular areas and meninges; 2, mild cellular infiltrate in parenchyma; 3, moderate cellular infiltrate in parenchyma; 4, severe cellular infiltrate in parenchyma. Myelin breakdown was assessed as pale staining with LFB and scored as follows: 0, no demyelination; 1, mild demyelination; 2, moderate demyelination; 3, severe demyelination.

For immunostaining, CNS paraffin sections (5 µm) were dewaxed and rehydrated treated with standard antigen retrieval protocol (0.01 M citrate buffer [20]). Endogenous peroxidase activity was quenched by adding 0.6% H_2_O_2_. Non-specific binding was minimized by CAS protein blocking solution (Invitrogen). Sections were incubated with anti-CD3 (Abcam) or anti-F4/80 (eBioscience) antibodies. Appropriate secondary antibodies were used and detected using DAB (Vector Laboratories). Slides were counterstained with hematoxylin. Three spinal cord sections were analyzed per mouse.

### hAEC tracking

After harvesting, primary hAEC from n = 4 donors were pooled and labeled with CFSE (Invitrogen) as described previously [19]. Briefly, CFSE was dissolved in DMSO and further diluted 1000 times with DMEM-F12, then added to the hAEC suspension (1×10^6^ cells/ml) at a final concentration of 10 µM. After incubation at 37°C for 10 min, the staining was quenched by addition of 5 volumes of DMEM/F12. The CFSE-labeled hAEC were then washed, resuspended in DMEM-F12 medium and intravenously injected into MOG immunized mice (n = 5). Each mouse received 2×10^6^ cells in 200 µl medium. Seven days later, mice were killed and organs were collected. Single cells from 1/3 of each spleen and the right inguinal lymph node were analyzed by acquiring 1.5–2×10^6^ live cell events using flow cytometry while the remaining spleens, the left inguinal lymph nodes, lungs, the livers, the brains and spinal cords were frozen in OCT and sectioned (5 µm thick) and examined for CFSE positive cells. Between 9–16 frozen tissue sections were analyzed for each organ/mouse.

### Statistical Analysis

Data are presented as mean±SEM and evaluated by one-way ANOVA with Tukey's test or Student's T-test. A *P* value <0.05 was considered significant.

## Results

### hAEC suppress *in vitro* splenocyte proliferation

**Figure 1:**
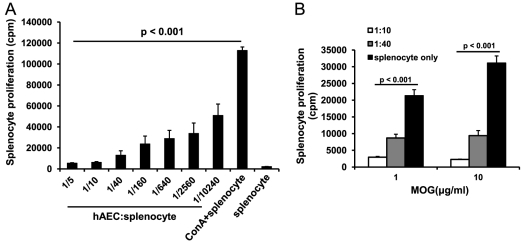
Dose-dependent suppression of splenocyte proliferation by hAEC. (**A**) ConA (5 µg/ml) stimulated proliferation of splenocytes from naive mice was dose-dependently inhibited by hAEC in hAEC∶splenocyte ratios ranging from 1∶5 to 1∶10240 (n = 5). (**B**) Proliferation of splenocytes from EAE mice stimulated by 1 µg/ml and 10 µg/ml MOG peptide was inhibited by hAEC (n = 3) at hAEC∶splenocyte ratios of 1∶10 and 1∶40. Splenocyte proliferation is expressed as counts per minute (cpm) of ^3^H-thymidine incorporation. All data are means±SEM.

Since T cell-mediated responses are thought to be crucial for MS development, we assessed hAEC for their capacity to suppress T cell proliferation *in vitro* and then assessed their potential to relieve MS-like symptoms in a mouse model *in vivo*. We found that hAEC potently suppressed the proliferation of splenocytes from naive mice stimulated with 5 µg/ml ConA. Dose-dependent suppression of ConA stimulation of splenocytes was observed at hAEC∶splenocyte ratios ranging from 1∶5 to 1∶10240. Suppression of 95% was observed at hAEC∶splenocyte ratios of 1∶5 and 1∶10, and even at hAEC∶splenocyte ratio of 1∶10240 suppression of 55% was still observed (Fig. 1A). hAEC also exerted similar immunosuppressive effects in a MOG antigen-specific setting. Splenocytes from MOG-induced diseased mice showed vigorous proliferation to MOG peptide re-stimulation (Fig. 1B). hAEC potently inhibited these proliferative responses by 60–90% at hAEC∶splenocyte ratios of 1∶10 and 1∶40 at different MOG peptide concentrations (1 and 10 µg/ml) (Fig. 1B). Thus, hAEC exert potent suppression of splenocyte proliferation in both antigen non-specific and antigen-specific manner.

### hAEC ameliorate EAE and improve CNS histopathology

**Figure 2:**
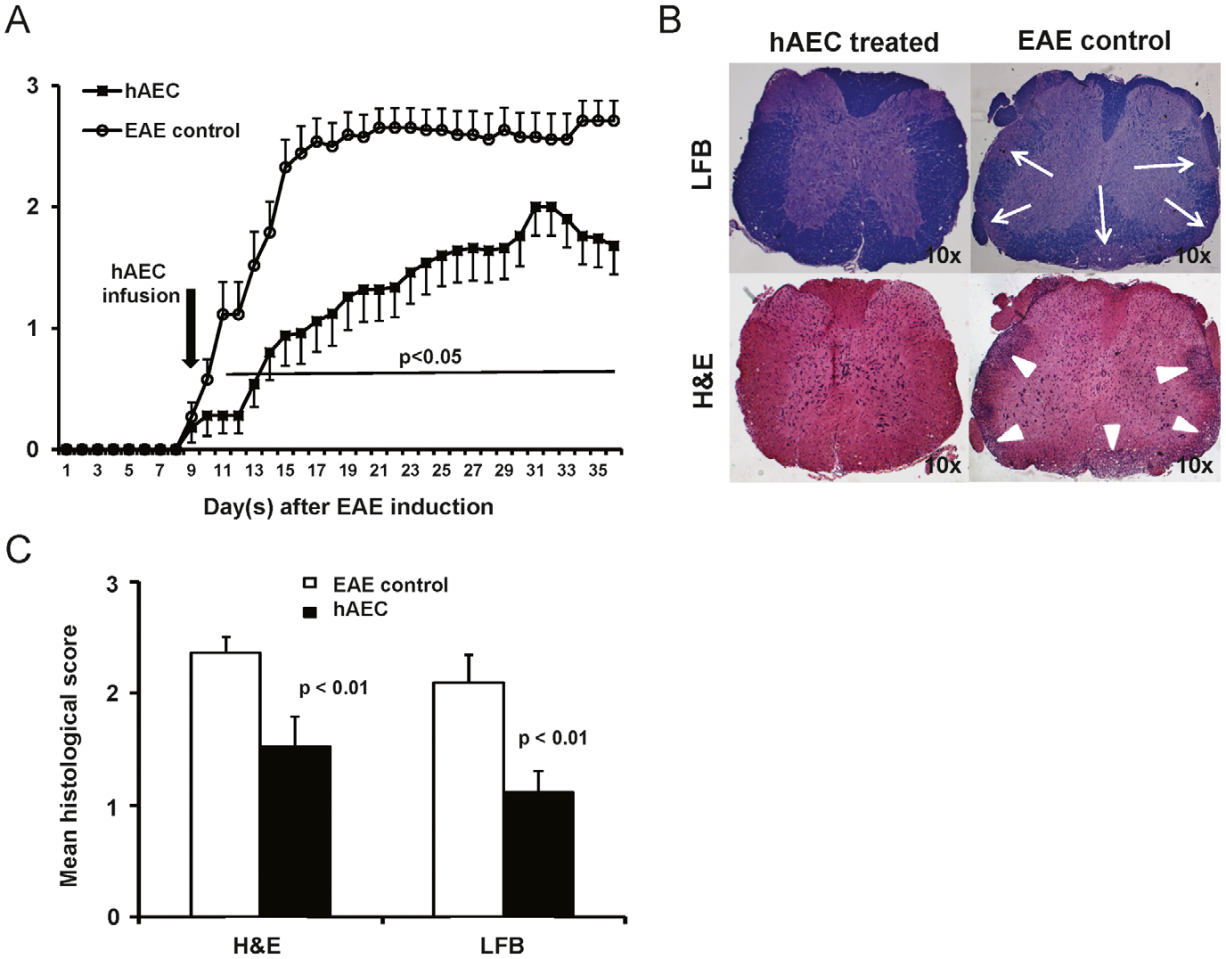
hAEC infusion potently ameliorated EAE and reduced spinal cord pathology. EAE was induced by immunization with 200 µg MOG_35–55_ peptide in 100 µl CFA followed by 350 ng pertussis toxin and the clinical scores were evaluated. (**A**) hAEC (2×10^6^) injected intravenously on day 9 after EAE induction ameliorated disease development (n = 25 in total) while control animals developed EAE (n = 26 in total). Data shown are combined results of three independent experiments each using pooled hAEC from n = 4 donors. (**B**) Spinal cord sections were stained by H&E and LFB for assessment of cellular infiltrates and demyelination, respectively. Representative spinal cord sections from hAEC-treated mice showed intact myelin sheath (blue color) from LFB staining and no cellular infiltrate from H&E staining. EAE control sections show regions of demyelination (arrows) and cellular infiltrate (arrow heads). (**C**) hAEC-treated mice (n = 9) showed significantly lower histological scores in both H&E and LFB assessments compared to EAE control (n = 5). All data are means±SEM.

Given the potent immunosuppressive effect of hAEC *in vitro*, we then assessed their *in vivo* therapeutic effects by injecting 2×10^6^ hAEC into mice at day 9 post-EAE induction. hAEC infusion ameliorated EAE in mice from three independent experiments with significance detected in mean scores after day 11 (p<0.05). The hAEC-treated mice (n = 25) had mild or delayed EAE compared to their littermate controls (n = 26, Fig. 2A).

**Figure 3:**
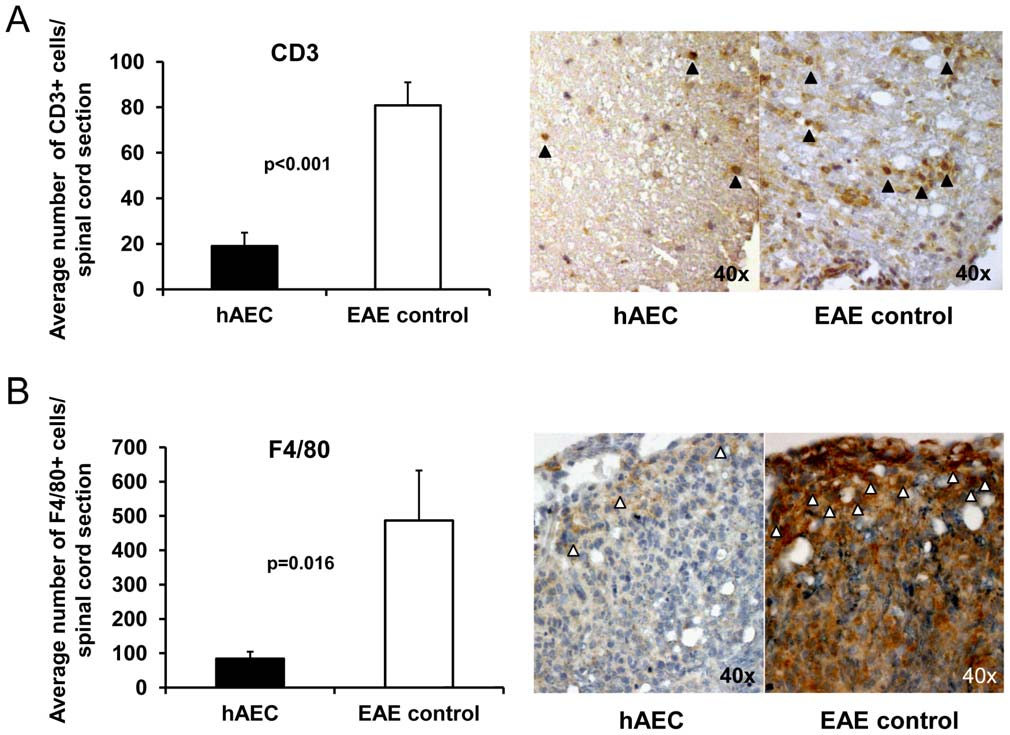
Reduced CD3^**+**^ T cells and F4/80^**+**^ monocytes/macrophages in CNS. Average number of CD3^+^ T cells (**A**) and F4/80^+^ cells (**B**) enumerated per spinal cord section (mean of 3 sections per mouse) showed significant reduction in hAEC-treated mice (n = 8) compared to EAE control (n = 4). Photomicrographs showing representative CD3 (black arrowheads) and F4/80 (white arrowheads) staining in hAEC-treated and EAE control spinal cords. All data are means±SEM.

Examination of spinal cords from hAEC-treated mice showed no or minimal inflammatory cell infiltration and myelin loss while EAE control mice showed extensive cellular infiltration and demyelination (Fig. 2B). Blinded quantitation of these parameters via a validated histological scoring system [6], [21] showed that these differences in cellular infiltration and demyelination were significant (p<0.01; Fig. 2C). Inflammatory infiltrates such as T cells and monocytes into CNS play important roles in the pathogenesis of EAE [1], [2], [22]. Thus, we further investigated the cellular infiltrate in the spinal cords for the presence of inflammatory cells. We found significant reduction in the numbers of CD3^+^ T cells (p<0.001; Fig. 3A) and F4/80^+^ monocytes/macrophages (p = 0.016; Fig. 3B) in the spinal cords from hAEC- treated mice compared to controls.

### Clinical improvement is associated with decreased immune response to MOG and a Th2 shift

**Figure 4:**
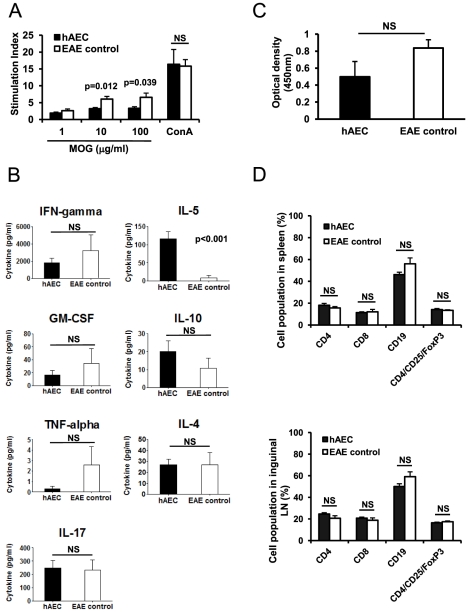
hAEC-treated mice showed reduced splenocyte proliferation and Th2 cytokine shift following stimulation *in vitro* with MOG peptide but serum MOG antibody and lymphocyte populations in spleen and lymph nodes remained unaffected. (**A**) Splenocytes from MOG-immunized and hAEC-treated mice (n = 8) stimulated with MOG peptide proliferated significantly lower than EAE control mice (n = 5) at 10 and 100 µg/ml MOG peptide. ConA (10 µg/ml) stimulated proliferation from both groups was similar. (**B**) Cytokines in supernatants from splenocyte cultures in (A) stimulated with 10 µg/ml of MOG for 72 hr were analyzed. IL-5 was significantly elevated. (**C**) MOG-specific antibodies in sera of hAEC-treated mice (n = 8) and EAE control (n = 5) were not significantly different. (**D**) FACS analysis of T cell subsets (CD4^+^, CD8^+^, CD4^+^CD25^+^ FoxP3^+^) and B cells (CD19^+^) from the spleen (upper panel) and from inguinal lymph node (lower panel) showed no significant differences between hAEC-treated mice (n = 9) and EAE control (n = 5). All data are means±SEM. NS: Not significant.

Since hAEC potently suppressed lymphocyte-mediated EAE development, we tested whether lymphocytes from hAEC-treated mice had reduced responsiveness to *ex vivo* MOG stimulation. We found that splenocytes from hAEC-treated mice proliferated significantly less than EAE control mice after MOG peptide stimulation (10 and 100 µg/ml), but their antigen non-specific proliferation to the mitogen ConA (10 µg/ml) remained similar (Fig. 4A). Splenocytes from hAEC-treated mice appeared to produce less Th1 cytokine IFN-gamma and less inflammatory cytokines GM-CSF and TNF-α; although the reduction did not reach statistical significance (Fig. 4B). In contrast, the Th2 cytokines IL-5 and IL-10 appeared increased with the increase in IL-5 reaching a significance of p<0.001; however, IL-4 remained unchanged. There was also no significant difference in IL-17 levels (Fig. 4B). We also examined the effect of hAEC on B cells by measuring anti-MOG antibody in serum from hAEC-treated mice and from EAE control mice. hAEC treatment led to a reduction in MOG-specific autoantibodies in sera but it failed to reach statistical significance (Fig. 4C).

To determine whether hAEC transplantation altered the proportion of T and B lymphocyte subpopulations in peripheral lymphoid organs, we analyzed cells from the spleen and inguinal lymph node by flow cytometry. We showed that there were no significant differences in the percentages of CD4^+^, CD8^+^, CD19^+^, and CD4^+^CD25^+^FoxP3^+^ [23], [24], [25] lymphocyte sub-populations between hAEC-treated and the control group (Fig. 4D).

### hAEC utilize TGF-β and PGE2 for immunosuppression

**Figure 5:**
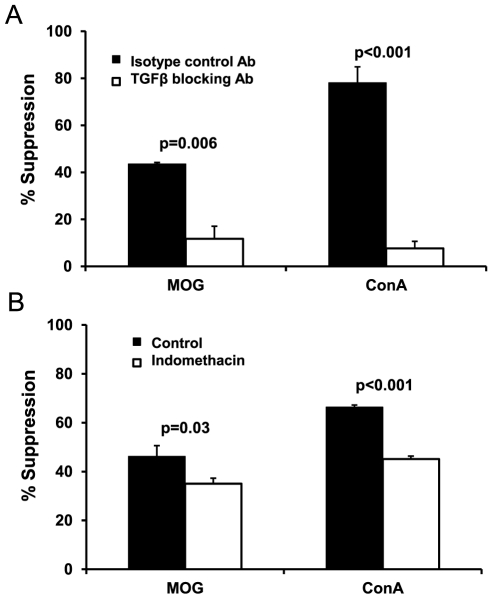
TGF-β blocking antibody and indomethacin reversed hAEC suppression of splenocyte proliferation stimulated by ConA and MOG peptide. hAEC∶splenocyte at 1∶40 ratio were co-cultured with splenocytes from EAE mice and MOG (10 µg/ml), or with splenocytes from naïve mice and ConA (5 µg/ml). Data shown is from n = 3 experiments each using pooled cells from n = 4 donors. Addition of TGF-β neutralizing antibody (**A**) or PGE2 antagonist indomethacin (**B**) significantly reduced the suppression exerted by hAEC in both settings. Data presented as percentage of suppression compared with TGF-β neutralizing antibody or PGE2 antagonist treated control groups (splenocytes+MOG/ConA), respectively. All data are means±SEM.

Soluble factors have been suggested to be utilized by hAEC to modulate immune responses [15]. We detected TGF-β1 (86.09±26.66 pg/ml/million hAEC) and PGE2 (85.33±30.55 pg/ml/million hAEC) in serum-deprived hAEC-conditioned culture medium but were unable to detect HGF, NO or IL-10. The result suggested that TGF-β and PGE2 might be important factors produced by hAEC that suppressed the proliferation of stimulated splenocytes. Thus, we set up two co-culture settings using hAEC and splenocytes from EAE-diseased mice stimulated with MOG peptide or from naïve mice stimulated with ConA. We added neutralizing antibody to block TGF-β or indomethacin to inhibit PGE2 in the co-culture. The presence of TGF-β neutralizing antibody or indomethacin significantly abrogated the inhibition of splenocyte proliferation by hAEC in either MOG peptide or ConA-stimulated cultures (Fig. 5A, B). Blocking of TGF-β reduced the suppressive effects on MOG-stimulated splenocytes by nearly 75% and almost 90% on ConA-stimulated splenocytes. Similarly, blocking of PGE2 by indomethacin reduced the suppression of MOG-stimulated splenocytes and of ConA-stimulated splenocytes by approximately 20%. Thus, these data confirmed that TGF-β and PGE2 are crucial for hAEC-mediated suppression *in vitro*.

### Detection of CFSE-labeled hAEC

**Figure 6:**
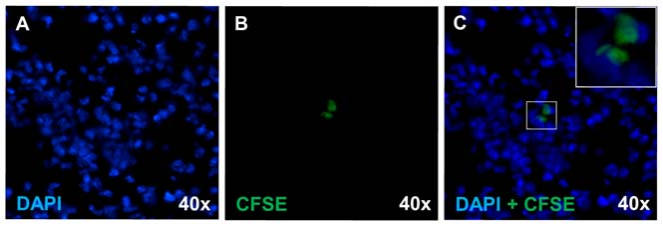
Tracking of CFSE-labeled hAEC. Lung section obtained from mice injected with CFSE-labeled hAEC after MOG immunization and organ collected 7 days later (5 mice for each group, 9–16 sections per organ were examined). (**A**) Frozen lung section staining with DAPI. (**B**) Same field as (A) showing CFSE positive cells. (**C**) Overlay of (A) and (B). Insert showing enlarged view of CFSE-DAPI positive cells.

Given the beneficial clinical outcome of hAEC infusion on EAE development, we further investigated the possible locations of these cells in their mouse recipient. We injected CFSE-labeled hAEC and tracked them in peripheral organs and CNS of hAEC-treated mice, and detected CFSE positive cells in lung tissues 7 days after cell administration (Fig. 6). On average, 2–5 CFSE positive cells were detected per 5 µm thick lung section. However, we failed to detect CFSE positive cells from spleen, inguinal lymph node, liver, brain and spinal cord sections (data not shown). In addition, flow cytometric analysis of spleens and inguinal lymph nodes yielded the same results as frozen sections.

## Discussion

This is the first study to report the therapeutic effect of hAEC in a mouse model of multiple sclerosis and that TGF-β is utilized by hAEC for immunosuppression. hAEC transplantation has previously been reported to ameliorate fibrosis models in the liver and in the lung [20], [26]. These cells have also ameliorated a Parkinson's disease model by increased production of neurotrophic factors that enhanced local repair [27]. Our study demonstrates that hAEC have potent immunosuppressive ability both *in vitro* and *in vivo*. In ConA-stimulated and MOG-stimulated hAEC-splenocyte co-culture assays, hAEC exerted suppression in both antigen non-specific and antigen-specific settings. Furthermore, hAEC infusion on day 9 after EAE induction protected mice from disease development.

We identified TGF-β and PGE2 as molecules utilized by hAEC for immunosuppression. TGF-β, a T cell growth inhibitor which inhibits T cell proliferation and DNA synthesis [28], [29], [30], is a powerful immunosuppressive molecule. TGF-β deficient mice suffer severe multifocal inflammatory lesions [31] while blocking TGF-β signaling in T cells causes disruption in T cell development and function [32]. TGF-β signaling in dendritic cells is also needed to control autoreactive myelin-reactive T cells [33]. TGF-β is thought to promote the resolution of inflammation with systemic treatment suppressing CNS inflammatory lesions and signs of EAE [34], [35]. In a previous study, using human specific PCR primers we have shown that TGF-β mRNA is expressed in lungs of hAEC-treated mice [26], and therefore it is likely that TGF-β produced by hAEC contributed to the improvement of EAE in the current study. On the other hand, PGE2 has a variety of immunosuppressive properties including inhibition of T cell proliferation and stimulating the production of Th2 cytokines such as IL-5 and IL-10 [36], [37]. The actions of these molecules are in agreement with our observations that splenocytes from hAEC-treated, EAE-protected mice, proliferated significantly less when stimulated *ex vivo* with MOG, and that supernatants from these cultures revealed a Th2 shift in their cytokine profile with significantly elevated IL-5. Upregulation of Th2 cytokines such as IL-5 have been shown to have a protective effect on EAE [38], [39].

Multiple sclerosis and EAE are considered T cell-mediated diseases [1], [2] because adoptive transfer of CNS antigen-activated T cells is sufficient to induce EAE [40], [41]. Activation of CNS-reactive T cells initiates local microglia expansion and recruitment of blood-borne monocytic cells [42], [43]. These cells secrete pro-inflammatory cytokines and participate in demyelination [44]. Monocytic infiltration into the CNS is correlated with progression of clinical disease and blocking their infiltration prevents EAE progression [22]. Thus, the significant milder EAE and reduced demyelination in hAEC-treated mice are in agreement with our observations of dramatic reduction in CD3^+^ T cell and F4/80^+^ monocyte/macrophage infiltration in the CNS.

Taken together, our findings suggest that by producing TGF-β and PGE2, hAEC treatment limited the expansion of MOG-reactive T cells and their infiltration into the CNS which in turn limited monocytic cell infiltration, and disease development. In addition, the reported neurotrophic factors such as brain-derived neurotrophic factor and nerve growth factor [16] produced by hAEC may also have contributed in part to the beneficial effect in hAEC-treated EAE mice.

We attempted to track the whereabouts of the systemic delivered hAEC. Seven days after the injection of hAEC into EAE-induced mice, we could only detect CFSE-labeled hAEC in the lungs without detectable cells in CNS or peripheral lymphoid organs. From the mesenchymal stem/stromal cell (MSC) literature, MSC were found to provide clinical benefits without their necessary presence in the affected organs, with most of the cells trapped within the lungs [45], [46]. Instead, it is now being proposed that MSC act from a distance by producing immunomodulatory molecule(s) during their transient appearance after systemic injection. Whether a similar scenario applies to hAEC awaits further investigation.

Our study demonstrated that hAEC have potent immunosuppressive capacity in the murine model of MS both *in vitro* and *in vivo*. Given the ready availability of human placenta and the ease of hAEC isolation from this organ, we suggest that hAEC has potential for application as cell therapy for multiple sclerosis. However studies using fewer numbers of hAEC in the mouse model and more information on the immunomodulative effects of hAEC in humans should be acquired before consideration can be given for clinical application in MS.

## References

[1] Hafler DA, Slavik JM, Anderson DE, O'Connor KC, De Jager P (2005). Multiple sclerosis.. Immunol Rev.

[2] Stinissen P, Raus J, Zhang J (1997). Autoimmune pathogenesis of multiple sclerosis: role of autoreactive T lymphocytes and new immunotherapeutic strategies.. Crit Rev Immunol.

[3] Clements CS, Reid HH, Beddoe T, Tynan FE, Perugini MA (2003). The crystal structure of myelin oligodendrocyte glycoprotein, a key autoantigen in multiple sclerosis.. Proc Natl Acad Sci U S A.

[4] von Budingen HC, Tanuma N, Villoslada P, Ouallet JC, Hauser SL (2001). Immune responses against the myelin/oligodendrocyte glycoprotein in experimental autoimmune demyelination.. J Clin Immunol.

[5] Slavin A, Ewing C, Liu J, Ichikawa M, Slavin J (1998). Induction of a multiple sclerosis-like disease in mice with an immunodominant epitope of myelin oligodendrocyte glycoprotein.. Autoimmunity.

[6] Chan J, Ban EJ, Chun KH, Wang S, Backstrom BT (2008). Transplantation of bone marrow transduced to express self-antigen establishes deletional tolerance and permanently remits autoimmune disease.. J Immunol.

[7] Chan J, Ban EJ, Chun KH, Wang S, McQualter JL (2008). Methylprednisolone induces reversible clinical and pathological remission and loss of lymphocyte reactivity to myelin oligodendrocyte glycoprotein in experimental autoimmune encephalomyelitis.. Autoimmunity.

[8] Uccelli A, Laroni A, Freedman MS (2011). Mesenchymal stem cells for the treatment of multiple sclerosis and other neurological diseases.. Lancet Neurol.

[9] Gang EJ, Bosnakovski D, Figueiredo CA, Visser JW, Perlingeiro RC (2007). SSEA-4 identifies mesenchymal stem cells from bone marrow.. Blood.

[10] Ilancheran S, Michalska A, Peh G, Wallace EM, Pera M (2007). Stem cells derived from human fetal membranes display multilineage differentiation potential.. Biol Reprod.

[11] Miki T, Lehmann T, Cai H, Stolz DB, Strom SC (2005). Stem cell characteristics of amniotic epithelial cells.. Stem Cells.

[12] Parolini O, Alviano F, Bagnara GP, Bilic G, Buhring HJ (2008). Concise review: isolation and characterization of cells from human term placenta: outcome of the first international Workshop on Placenta Derived Stem Cells.. Stem Cells.

[13] Bailo M, Soncini M, Vertua E, Signoroni PB, Sanzone S (2004). Engraftment potential of human amnion and chorion cells derived from term placenta.. Transplantation.

[14] Wolbank S, Peterbauer A, Fahrner M, Hennerbichler S, van Griensven M (2007). Dose-dependent immunomodulatory effect of human stem cells from amniotic membrane: a comparison with human mesenchymal stem cells from adipose tissue.. Tissue Eng.

[15] Li H, Niederkorn JY, Neelam S, Mayhew E, Word RA (2005). Immunosuppressive factors secreted by human amniotic epithelial cells.. Invest Ophthalmol Vis Sci.

[16] Uchida S, Inanaga Y, Kobayashi M, Hurukawa S, Araie M (2000). Neurotrophic function of conditioned medium from human amniotic epithelial cells.. J Neurosci Res.

[17] Uchida S, Suzuki Y, Araie M, Kashiwagi K, Otori Y (2003). Factors secreted by human amniotic epithelial cells promote the survival of rat retinal ganglion cells.. Neurosci Lett.

[18] Miki T, Marongiu F, Ellis E, S CS (2007). Isolation of amniotic epithelial stem cells.. Curr Protoc Stem Cell Biol Chapter.

[19] Pratama G, Vaghjiani V, Tee JY, Liu YH, Chan J (2011). Changes in culture expanded human amniotic epithelial cells: implications for potential therapeutic applications.. PLoS ONE.

[20] Manuelpillai U, Tchongue J, Lourensz D, Vaghjiani V, Samuel CS (2010). Transplantation of human amnion epithelial cells reduces hepatic fibrosis in immunocompetent CCl-treated mice.. Cell Transplant.

[21] Chen XT, Chan ST, Hosseini H, Layton D, Boyd R (2011). Transplantation of retrovirally transduced bone marrow prevents autoimmune disease in aged mice by peripheral tolerance mechanisms.. Autoimmunity.

[22] Ajami B, Bennett JL, Krieger C, McNagny KM, Rossi FM (2011). Infiltrating monocytes trigger EAE progression, but do not contribute to the resident microglia pool.. Nat Neurosci.

[23] Sakaguchi S, Miyara M, Costantino CM, Hafler DA (2010). FOXP3+ regulatory T cells in the human immune system.. Nat Rev Immunol.

[24] Wing K, Sakaguchi S (2010). Regulatory T cells exert checks and balances on self tolerance and autoimmunity.. Nat Immunol.

[25] Workman CJ, Szymczak-Workman AL, Collison LW, Pillai MR, Vignali DA (2009). The development and function of regulatory T cells.. Cell Mol Life Sci.

[26] Moodley Y, Ilancheran S, Samuel C, Vaghjiani V, Atienza D (2010). Human amnion epithelial cell transplantation abrogates lung fibrosis and augments repair.. Am J Respir Crit Care Med.

[27] Kong XY, Cai Z, Pan L, Zhang L, Shu J (2008). Transplantation of human amniotic cells exerts neuroprotection in MPTP-induced Parkinson disease mice.. Brain Res.

[28] Kehrl JH, Wakefield LM, Roberts AB, Jakowlew S, Alvarez-Mon M (1986). Production of transforming growth factor beta by human T lymphocytes and its potential role in the regulation of T cell growth.. J Exp Med.

[29] McKarns SC, Kaminski NE (2000). TGF-beta 1 differentially regulates IL-2 expression and [3H]-thymidine incorporation in CD3 epsilon mAb- and CD28 mAb-activated splenocytes and thymocytes.. Immunopharmacology.

[30] Shalaby MR, Ammann AJ (1988). Suppression of immune cell function in vitro by recombinant human transforming growth factor-beta.. Cell Immunol.

[31] Shull MM, Ormsby I, Kier AB, Pawlowski S, Diebold RJ (1992). Targeted disruption of the mouse transforming growth factor-beta 1 gene results in multifocal inflammatory disease.. Nature.

[32] Li MO, Sanjabi S, Flavell RA (2006). Transforming growth factor-beta controls development, homeostasis, and tolerance of T cells by regulatory T cell-dependent and -independent mechanisms.. Immunity.

[33] Laouar Y, Town T, Jeng D, Tran E, Wan Y (2008). TGF-beta signaling in dendritic cells is a prerequisite for the control of autoimmune encephalomyelitis.. Proc Natl Acad Sci U S A.

[34] Racke MK, Cannella B, Albert P, Sporn M, Raine CS (1992). Evidence of endogenous regulatory function of transforming growth factor-beta 1 in experimental allergic encephalomyelitis.. Int Immunol.

[35] Racke MK, Dhib-Jalbut S, Cannella B, Albert PS, Raine CS (1991). Prevention and treatment of chronic relapsing experimental allergic encephalomyelitis by transforming growth factor-beta 1.. J Immunol.

[36] Harris SG, Padilla J, Koumas L, Ray D, Phipps RP (2002). Prostaglandins as modulators of immunity.. Trends Immunol.

[37] Woolard MD, Wilson JE, Hensley LL, Jania LA, Kawula TH (2007). Francisella tularensis-infected macrophages release prostaglandin E2 that blocks T cell proliferation and promotes a Th2-like response.. J Immunol.

[38] Li H, Nourbakhsh B, Safavi F, Li K, Xu H (2011). Kit (W-sh) mice develop earlier and more severe experimental autoimmune encephalomyelitis due to absence of immune suppression.. J Immunol.

[39] Zhang GX, Yu S, Gran B, Rostami A (2005). Glucosamine abrogates the acute phase of experimental autoimmune encephalomyelitis by induction of Th2 response.. J Immunol.

[40] Engelhardt B (2006). Molecular mechanisms involved in T cell migration across the blood-brain barrier.. J Neural Transm.

[41] Zamvil SS, Steinman L (1990). The T lymphocyte in experimental allergic encephalomyelitis.. Annu Rev Immunol.

[42] Hickey WF, Hsu BL, Kimura H (1991). T-lymphocyte entry into the central nervous system.. J Neurosci Res.

[43] Swanborg RH (1995). Experimental autoimmune encephalomyelitis in rodents as a model for human demyelinating disease.. Clin Immunol Immunopathol.

[44] Benveniste EN (1997). Role of macrophages/microglia in multiple sclerosis and experimental allergic encephalomyelitis.. J Mol Med (Berl).

[45] Prockop DJ, Youn Oh J (2011). Mesenchymal Stem/Stromal Cells (MSCs): Role as Guardians of Inflammation.. Mol Ther.

[46] Roddy GW, Oh JY, Lee RH, Bartosh TJ, Ylostalo J (2011). Action at a Distance: Systemically Administered Adult Stem/Progenitor Cells (MSCs) Reduce Inflammatory Damage to the Cornea Without Engraftment and Primarily by Secretion of TNF-alpha Stimulated Gene/Protein 6.. Stem Cells.

